# Community health workers in clinical research at the example of a phase IIIb/ IV antimalarial drug trial conducted in five African countries

**DOI:** 10.1016/j.ijid.2023.10.016

**Published:** 2023-12

**Authors:** Mirjam Groger, Gaston Tona Lutete, Serge-Brice Assi, Jude D. Bigoga, Nsengi Y. Ntamabyaliro, Sarah Arbe-Barnes, Jangsik Shin, Ayola A. Adegnika, Francine Ntoumi, Peter G. Kremsner, Michael Ramharter, Stephan Duparc, Isabelle Borghini-Fuhrer, Ghyslain Mombo-Ngoma

**Affiliations:** 1 Institut für Tropenmedizin, University of Tübingen, Tübingen, Germany; 2 Center of Tropical Medicine, Bernhard Nocht Institute for Tropical Medicine and I. Department of Internal Medicine I, University Medical Center Hamburg-Eppendorf, Hamburg, Germany; 3 Unité de Pharmacologie Clinique et Pharmacovigilance (UPC-PV), University of Kinshasa, Kinshasa, Democratic Republic of Congo; 4 Institut Pierre Richet/Institut National de Santé Publique (IPR/INSP), Bouaké, Côte d'Ivoire; 5 The Biotechnology Center, University of Yaoundé I, Yaoundé, Cameroon; 6 Artemida Pharma, Stevenage, SG1 2FX United Kingdom; 7 Shin Poong Pharmaceutical, Seoul, Republic of Korea; 8 Centre de Recherches Médicales de Lambaréné (CERMEL), Lambaréné, Gabon; 9 German Center for Infection Research (DZIF), Tübingen, Germany; 10 Fondation Congolaise pour la Recherche Médicale (FCRM), WHO-AFRO Campus Djoué, Brazzaville, Hospital of Talangai (North) of Brazzaville, Republic of Congo; 11 Medicines for Malaria Venture, Geneva, Switzerland; 12 Department of Implementation Research, Bernhard Nocht Institute for Tropical Medicine and I. Department of Internal Medicine I, University Medical Center Hamburg-Eppendorf, Hamburg, Germany

**Keywords:** Community health worker, Pyronaridine-artesunate, Rethink malaria, CANTAM

## Abstract

Global health, particularly in underserved settings can benefit immensely from well-trained community health workers (CHWs) supporting primary healthcare interventions. They can reduce morbidity and mortality of infectious diseases like malaria. Disease control programs can particularly benefit from a tight link between CHWs and communities and several studies have shown the benefit of the participation of non-facility-based CHWs in malaria control program activities for reducing malaria-related mortality in children. Because CHWs are often part of and trusted by served communities, they can also be an important resource to address challenges faced by their communities. Where post-marketing surveillance systems are underserved, they can relay important information about suspected safety signals and factors affecting therapeutic effectiveness in their communities. The CANTAM-Pyramax® trial was a phase IIIb/ IV cohort event monitoring study conducted at six centers in five African countries. To assess real-world effectiveness and safety of the anti-malarial pyronaridine-artesunate in 8560 malaria episodes, follow-up was not primarily conducted by medical staff but by specifically trained CHWs. This perspective paper discusses how the participation of a CHW workforce can be of benefit for effectiveness trials in limited-resource settings, using the example of the CANTAM-*Pyramax* trial.

## Highlights

• Use of controlled research methods in real-life conditions.
• Community health workers can diversify patient populations in clinical research.
• Assessment of real-world effectiveness can improve generalizability of results.

## Malaria elimination and control over the past decades

Malaria is a parasitic disease transmitted by infected *Anopheles* mosquitoes. Despite the over a century-long history of control and elimination efforts, malaria still represents an important disease burden, particularly for sub-Saharan Africa [1]. New tools and interventions like insecticide-treated bed nets or the introduction of artemisinin-based combination therapies have contributed importantly to the progress toward malaria elimination and eradication since the beginning of the millennium. Notwithstanding, this progress has stalled since 2015 and the 2020 milestones of the “Global Technical Strategy For Malaria” set forth by the World Health Organization (WHO) could not be reached [2]. While elimination could be accomplished in several countries with low endemicity, absolute case numbers have further increased in the 10 highest burden countries in Africa [3].

## Rethinking malaria

High- and low-burden countries require different approaches to increase the impact of malaria-related interventions. Value-based health services delivery is a relatively new key term for sub-national tailoring of malaria control strategies in line with the needs of local communities [4]. Rethinking the approach with which to combat malaria and integrating malaria response strategies into primary health care becomes one of the WHO's key strategies. Those newly identified strategies include multi-sectoral approaches, the increase of country ownership in the development of malaria control strategies, in-country evaluation and interpretation of community-level data with informed strategic policymaking, and integrated management approaches at community level [3,[5], [6], [7]]. Community health workers (CHWs) and communities themselves gain center stage to share their experiences with malaria as those directly affected and contribute to identifying tailored solutions for their communities [5].

## CHW as an extension of health care- and surveillance systems

An important factor contributing to preventable malaria morbidity and mortality, as well as morbidity and mortality from other preventable diseases is a lack of access to the healthcare system [8]. In many communities, CHWs provide public health interventions and form an important link between underserved communities and the healthcare system [9]. The integrated community case management (iCMM) strategy seeks to train CHWs to enable them to provide care for a range of priority health issues in a community [10,11]. With limited to no access to facility-based nurses or doctors, this can help reduce morbidity and mortality locally.

CHWs often are lay people trained on program-specific health care interventions. Due to their access to hard-to-reach communities, CHWs can contribute valuably to disease prevention and control and thus be an important addition to national malaria control programs [12]. Several studies have shown the benefit of the inclusion of non-facility-based CHWs in malaria control program activities for reducing malaria-related mortality in children [13]. CHWs can also support educational advertising and contribute to the progress toward the United Nations’ sustainable development goal number 3: “good health and well-being” by reaching large parts of a population despite a lack of trained healthcare personnel therein [14].

As CHWs are often embedded in and trusted by their communities, they can serve as resource for their community's challenges. In the absence of surveillance systems for post-marketing pharmacovigilance, they can feed back important information about drug safety and other relevant observations to regulators which can help identify and understand reduced effectiveness of therapies or safety concerns.

## Community health workers in clinical research

In research, CHWs can be deployed to improve post-hospital outcomes or adherence to routine medical interventions [15]. However, they can also benefit clinical drug development. Integrating CHWs in research teams has the potential to open up patient populations to become more diverse. (Ethnical) minorities, remote communities, and others that may otherwise not have easy access to health care, and may thus not be a target in passive case-finding approaches of clinical trial recruitment can become represented in a better way. This can result in more accurate data for drug development as the characteristics of the participant pool are closer to the true population characteristics.

An equally important basis for the successful conduct of clinical research projects is a trusting relationship between CHWs and communities. As CHWs often live in the communities they serve, CHW-centered recruitment may be facilitated by their inside knowledge of comorbidities, pregnancies, and other important medical histories of the individuals they are responsible for.

## Importance of adequate training

An important prerequisite for a functional collaboration with and inclusion of CHWs into the health care system is provision of adequate protocols and training [12,16]. The CHW can only be an expedient connecting link if thorough understanding of the matter is in place [17]. Sharing of misinformation, enforcement of superstition, or medical decision-taking outside the CHW's competence can corrupt the trusting relationship with communities as well as with healthcare personnel and harm patients [17,18]. In the framework of clinical trials, staff training is a key part, and supportive supervision, and re-training are often practiced throughout the project per default.

## The role of CHW in the CANTAM-*Pyramax* trial

The CANTAM-*Pyramax* trial was a phase IIIb/ IV cohort event monitoring study with the aim to assess the real-world safety, tolerability, and effectiveness of pyronaridine-artesunate (PA), an artemisinin-based combination treatment which is administered once daily over 3 consecutive days for the treatment of uncomplicated malaria. CHW-centered follow-up was a key strategy to determine real-world safety and effectiveness.

The trial was conducted at six centers in five African countries with the main objective to assess the safety of PA in malaria patients with underlying liver function abnormalities, comorbidities like HIV or viral hepatitis, and in children under 1 year of age [19]. These specific patient populations had not been included in the clinical development program until then, given the restrictive eligibility criteria for phase II and III trials. Patients with uncomplicated malaria, diagnosed in line with the standard of care in the respective country which was either a blood smear or rapid diagnostic test, were eligible to participate. The first treatment dose was administered under direct supervision, intake of subsequent doses was explained, and the participants continued self-treatment at home—as they would under routine care conditions. Follow-up home visits were conducted on day 7 ± 1 day and day 28 ± 2 days.

To evaluate safety and real-world effectiveness, participants were primarily followed up by CHWs trained specifically in the trial procedures. Depending on the country's strategies, most CHWs were recruited either from a pre-existing pool or through structured processes set up by e.g., the Ministry of Health. CHWs needed to be rapidly deployable and serve as a mobile link between the participants living at varying distances from the study centers and the investigator teams. They provided oversight of participant safety while intervening as little as possible with the participants’ healthcare-seeking behavior to mirror the real-world patient-doctor interactions in the management of uncomplicated malaria.

## Community health worker training and participant follow-up in the CANTAM-*Pyramax* trial

To neither jeopardize participants’ reporting behavior nor overlook important safety signals, continuous training was paramount for CHWs to remain alert of their responsibilities. Training was given during the site initiation visit and included an explanation of material and procedures as well as role play. Role plays were used to simulate real-life scenarios and discuss solutions. CHWs who had previously been involved in studies with home-based participant follow-up shared their experiences with the others. Re-trainings were provided by the site investigators during formalized training sessions or regular team meetings, and during monitoring visits as needed.

For self-training purposes, CHWs received a “memory aid” to carry with them for reference but they were not supposed to use it during direct participant contact to avoid any bias in participants’ reporting. The memory aid contained a general part, with a description of the seriousness criteria and the query whether treatment or medical attention was necessary. If any of the listed was answered with “yes”, the CHW was required to inform the investigator immediately and direct the participant to the study center to receive further medical attention. The second part of the memory aid focused on adverse events of special interest, these being hepatotoxicity and hypersensitivity. Warning signs for hepatotoxicity included fatigue of unusual intensity that remained after cure of the malaria episode or worsened; fever that either remained after cure of the malaria episode or returned after initial remission; nausea; vomiting; loss of appetite; stomachache; pruritus or skin rash. In addition, images of dark urine; putty or mastic colored stools; jaundice, and contusions, indicating spontaneous sub-cutaneous bleeding, were provided to facilitate CHWs in recognizing these signs. Hypersensitivity events were identified by any signs of flushing; the appearance of wheals/urticaria; breathlessness and faintness and/or decrease in blood pressure. CHWs found the memory aid to be a useful and self-explanatory tool.

To reflect real-life conditions as much as possible and avoid over-reporting of adverse events, suggestive questions were not permitted. Instead, the CHW was instructed to ask open questions and document (1) whether the participant noticed anything special or abnormal concerning their health status since the last contact with study staff or since the study drug intake and (2) whether the CHW noticed anything special or abnormal in the participant. In case the participant reported specific medical conditions or the CHW observed danger signs, they were referred to the study center for further investigation.

Home visits also included additional examinations within the CHW competence in the respective country. On day 7 ± 1 day, measurement of body temperature; enquiry of pregnancy status; assessment of compliance to study drug intake, and documentation of signs and symptoms of malaria and new and concomitant medication were performed. In addition, at day 28 ± 2 days, measurement of body temperature; pregnancy test where relevant; preparation of thick blood smear and blood spots on filter paper were conducted. Where CHWs were not allowed to collect blood, they were accompanied by a laboratory technician. If malaria or pregnancy was suspected, the CHW was instructed to refer the participant to the study center immediately.

Source documents for all home visits of the day, i.e., the standardized notes taken by the CHW, were reviewed by and discussed with the site investigator on the CHW's return to the study center. The investigator was responsible for the final judgment, documentation on the case report form, and further actions for adverse events. Based on this, if the investigator spotted the need for further medical evaluation or treatment, the participant was notified and instructed to attend the study center for further investigation. Malaria microscopy was performed on all thick blood smears at the center to assess response to the study treatment. In case a blood smear was positive participants were notified and directed to the study center to receive antimalarial rescue treatment.

Overall, CHWs appeared to be highly trusted by the target population and participants referred their peers to the CHWs for study participation when they were ill. This trust was largely based on the CHWs’ long-term activities in the communities and/or the fact that the CHWs were trusted community members. None of the study sites reported barriers when talking to participants. Challenges sometimes arose when participants lived far from the study site when participants’ residential areas were not well mapped, or when participants were not at home for scheduled visits. This was mitigated by calling participants, providing CHWs with cars or other means of transport, and being creative in describing participants’ homes (e.g., near the banana market, etc.).

## Other examples of community health workers-centered research

CHW-centered approaches are also being implemented by other research projects and surveillance programs. An example of a CHW-centered research project is the MiMBa Pregnancy Registry to assess the safety of antimalarial use in pregnancy [20]. This observational prospective study aims to generate robust evidence on the safety of a range of available and registered antimalarial when used in pregnancy, particularly in the first trimester, and fully depends on the CHW workforce. The CHWs are responsible for putting all subjects living in the study area into a comprehensive household registry. This first step makes it possible to subsequently cross-reference the registers of pregnant women with those of subjects who have contracted malaria to detect pregnant women who were treated with antimalarial drugs. A current example of a post-marketing surveillance program is the community access to rectal artesunate for malaria (CARAMAL) project [21]. Patients with severe malaria in remote settings often lack timely access to life-saving intravenous or intramuscular antimalarial therapy. An alternative solution, particularly for pediatric patients may be rectal artesunate suppository administered as part of iCCM until patients are well enough to switch to oral therapy. The roll-out of this therapeutic strategy is being included in iCCM platforms in selected African countries to ensure community access and to generate supporting surveillance data.

## Conclusion

CHW involvement can be key to targeting hard-to-reach and vulnerable populations and constitutes a crucial role in malaria control and elimination in sub-Saharan Africa. In the CANTAM-*Pyramax* trial, the CHW-centered approach provided a controlled methodology to generate reassuring data for regulators and health practitioners resulting in broader access of PA to patients [22]. By mimicking real-life conditions, the trial mirrored the reality of the field but within a controlled framework.

The CANTAM-*Pyramax* trial can serve as reference for the conduct of clinical trials and research projects focusing on the collection of real-world data, in alignment with the “rethinking malaria” initiative, but also in the context of other infectious diseases.

## Declarations of competing interest

IBF and SD are full-time employees at Medicines for Malaria Venture, JS is a full-time employee at Shin-Poong Pharmaceutical and SAB is a consultant paid by Shin-Poong Pharmaceutical. The other authors declare no conflict of interest.
